# Morphological and initial  molecular characterization of *Clogmia albipunctatus* larvae (Diptera: Psychodidae) causing urinary myiasis in Egypt

**DOI:** 10.1371/journal.pntd.0007887

**Published:** 2019-12-23

**Authors:** Haiam Mohammed Mahmoud Farrag, Enas Abdelhameed Mahmoud Huseein, Amal M. Almatary, Ragaa A. Othman

**Affiliations:** Medical Parasitology Department, Faculty of Medicine, Assiut University, Assiut, Egypt; National Institutes of Health, UNITED STATES

## Abstract

Myiasis is the infestation of human tissues by dipterous fly larvae of the class Insecta. *Clogmia albipunctatus*, family Psychodidae, is one of the most medically important insects that cause human myiasis. The aim of the present study is the morphological identification and the molecular characterization of moth flies causing many cases of urinary myiasis in Egypt, based on sequencing of the mitochondrial DNA of the larvae. Seven urinary samples of patients complaining of urinary symptoms and giving a history of low socioeconomic level were examined. Recovered larvae were identified using light microscopy and SEM. For molecular identification, the mitochondrial genes Cytochrome B (cytB), NADH1, NADH1, and 16S were sequenced and phylogenetically analyzed. The morphological and molecular characterization could accurately diagnose our patients to have *C*. *albipunctatus* infestation. Such results provided the initial set of data on the molecular identification and phylogenetic analysis of moth flies based on DNA barcoding in Egypt.

## Author summary

Myiasis is an infestation of human tissues and other vertebrates by dipterous fly larvae. Urogenital myiasis is a rare type of myiasis that occurs primarily in poor people with low socioeconomic levels. Accurate diagnosis of this rare form of myiasis is sometimes difficult and requires a detailed study of unfamiliar morphological patterns of the larvae. Based on combined morphological and molecular approaches, our results provided the first set of data on the molecular identification and phylogenetic analysis of moth flies, causing myiasis based on the DNA barcoding in Egypt. Such results present more understanding of causative species epidemiology, the vectorial potentiality, and the proper pest management practices.

## Introduction

Human myiasis is an illness caused by dipterans larvae infestation to vertebrates' tissues. There are many forms of myiasis as dermal, respiratory, nasopharyngeal, auricular, ophthalmic, gastric, rectal and intestinal [1]. Urogenital myiasis is a rare human type that usually occurring with poor sanitation and a lack of hygienic measures. It is usually associated with urinary manifestations such as dysuria, hematuria, and frequency of micturition [2].

Dipterous families commonly implicated in urogenital myiasis include Psychodidae, Sarcophagidae, Muscidae, Anisopodidae, Calliphoridae, and Scinoinidae [3]. Among family Psychodidae, *Psychoda alternate* and *Psychoda albipennis* are usually implicated [4]. *Clogmia albipunctata* which belongs to the same family, subfamily Psychodinae, has also been reported to cause myiasis in humans (e.g. nasopharyngeal, intestinal and urinary) [5][6][7]. Moreover, *C*. *albipunctatus* can elicit inhalant allergy as a result of inhaling fragments of their disintegrated body parts and play a significant role as a potential mechanical vector of nosocomial pathogens in German hospitals [8].

Members of subfamily Psychodinae are commonly present in the sites of buildings, like sewers, septic tanks, cesspools, sewage treatment plants, and other dark, moist places. Their females are non-blood suckers and they feed on sewage and moisture sources. Their larvae are coprophagous and saprophagous. They feed on decaying organic matter and the feces of the vertebrate. They are present in public places such as the bathrooms, sports centers, hotels, and hospitals, thus they are of public health and veterinary importance [9].

Psychoda fly is commonly known as moth flies. It is less than 5mm in length, dark in color with fuzzy, moth-like appearance. It has a hairy body with roof–like wings held over the body. Species identification depends mainly on the number and shape of antennal segments on the head of the adult [10]. Accurate diagnosis of this rare form of myiasis is sometimes difficult and requires a detailed study of the unfamiliar morphological patterns of the larvae [11]. In addition, the morphology of this larva is quite similar to that of many other insects such as Phlebotomidae, Muscidae, Culicidae, and Culicoides. Thus, the diagnosis remains of low sensitivity and more sensitive methods of detection should be applied [12].

Recently, many DNA-based analyses have been applied for the specific identification and characterization of many insect species. The sequences of the mitochondrial DNA have been successfully used for the identification of many species of insect groups [9]. Therefore, this study aimed to identify the morphological and the initial molecular characterization, with phylogenetic analysis, of moth flies causing urinary myiasis in Egypt, based on sequencing of the mitochondrial DNA of the flies.

## Material and methods

### Samples data

Seven urinary samples were referred from the Urology Department, Assiut University Hospital, for proper diagnosis, to Parasitological Lab of the Parasitology Department, Faculty of Medicine, Assiut University, Assiut, Egypt, in the period from March 2015 to September 2017.

They belonged to male and female patients whose ages ranged from five to twenty-four years and residents of various villages in Assiut Governorate, Egypt.

Patients were complaining of intermittent discharge of small, motile, dark-fleshy worm-like organisms in their voided urine, associated with dysuria, frequency of micturition and genital pruritus. However, there was no fever or hematuria. According to their sheets, they were of low socioeconomic level with poor hygienic conditions.

They had normal CBC, complete urine examination and urinary ultrasonography. Stool analysis with direct smear and formol ether concentration technique was conducted with no abnormalities.

After diagnosis, patients were directed to anthelminthic treatment in the form of two oral doses of ivermectin 100μg/Kg to the child patients and 200 μg/Kg for the adult patients and antibiotics were prescribed. We recommended following up patients for two months later.

### Collection and handling of larvae for entomologic study

Larvae were collected from each sample and were washed several times with saline. Collected larvae were kept in 70% ethyl alcohol for further examination and entomologic identification [11].

### For light microscopic examination

Collected larvae were incubated in transparent glass tubes containing 30% potassium hydroxide, after puncturing the larvae on the ventral sides (to dissolve the soft parts), till they became transparent under light microscope. Subsequently, the specimens were washed in distilled water, dehydrated in ascending grades of ethyl alcohol for 30min each, and were kept in xylene for 30min, then mounted in Canada balsam and dried in an oven at 38°C for 2 days [13]. The recovered larvae were examined under a light microscope and were photographed.

### For scanning electron microscope examination

The preserved larvae from each sample were washed carefully in distilled water, fixed for two hrs. in 4% glutaraldehyde and 5% paraformaldehyde in 0.1 M cacodylate buffer at a pH of 7.2. Subsequently, they were kept overnight in cacodylate buffer then dehydrated in graded aqueous ethanol 30%, 50%, 70% and 90% followed by critical point drying [14], sputter–coating specimens with gold in the sputter coating apparatus for six minutes. Finally, they were processed, examined and photographed in the unit of Scanning Electron Microscope of Assiut University by JEOL–JSM–5400 LV. The larvae were identified following the taxonomic identification key [15] and were illustrated by photomicrographs.

### For molecular identification

#### DNA extraction, amplification and sequencing:

Samples were preserved in 70% ethanol. The DNA was extracted using the QIAgen DNEasy Animal tissue extraction kit (Qiagen, Hombrechtikon, Switzerland). Two mitochondrial (hereafter mtDNA) fragments were amplified with two primer pairs as described by Simon et al. [16]: CB-J-11338 –CAC ATT CAA CCA GAA TGA TAT TT–and N1-N-12051 –GAT TTT GCT GAA GGT GAA TCA GA–for the first fragment and N1-J-12248 –AAG CTA ATC TAA CTT CAT AAG–and LR-N-12866 –ACA TGA TCT GAG TTC AAA CCG G–for the second fragment. The first fragment amplified portions of Cytochrome B (cytB) and NADH1 genes, while the second partially covered NADH1 and 16S genes.

The DNA samples were amplified by Thermocycler (veriti model 9902; Applied Biosystems, USA) using the following cycling protocol; For the first primer; one initial step of 95°C for 2.5 min, followed by 35 cycles of 30 sec at 95°C, 40 sec at 57°C, and 1 min at 72°C, in addition to a final step of 8 min at 72°C. For the second primer, the PCR condition was as follows: 95°C for 5 min, followed by 40 cycles of 1 min at 95°C, 1 min at 45°C, and 1.5 min at 72°C and a final single extension step of 10 min at 72°C [17].

The amplified products were analyzed by gel electrophoresis utilizing 1.5% agarose gel, stained with 0.05 μg mL^-1^ ethidium bromides (Serva, Germany) and visualized on a UV transilluminator. A 100 bp DNA ladder (Nippon Genetics Europe GmbH) was used to determine the size of the PCR products.

#### Sequencing and phylogenetic analysis:

The process of DNA sequencing was carried out in SolGent Company Limited (Daejeon, South Korea). The two amplified mtDNA fragments were sequenced using two primer pairs for each segment; CB-J-11338 –CAC ATT CAA CCA GAA TGA TAT TT–and N1-N-12051 –GAT TTT GCT GAA GGT GAA TCA GA–for the first fragment and N1-J-12248 –AAG CTA ATC TAA CTT CAT AAG–and LR-N-12866 –ACA TGA TCT GAG TTC AAA CCG G–for the second fragment, following the protocol reported by Espı´ndola et al. [17]. The obtained sequences were analyzed using the BLAST search program at the NCBI website: http://blast.ncbi.nlm.nih.gov/Blast.cgi. The alignment was assayed using the multiple sequence alignment program CLUSTALW. Phylogenetic analysis was performed with referential strains from GenBank MegAlign (ver. 5.01).

### Ethics statement

The experiment was conducted according to the principles expressed by the Committee for Ethics and Scientific Research of Faculty of Medicine, Assiut University which approved the acquisition of the samples. Written informed consents were obtained from adult patients and parents of the participating children to handle their samples and publish information about them.

### Accession numbers

The accession number of *Telmatoscopus albipunctatus* mitochondrial COI gene for cytochrome is AB907184.1, available at the NCBI’s LocusLink.

## Results

### Light microscopic description of full-grown larvae

**Fig 1:**
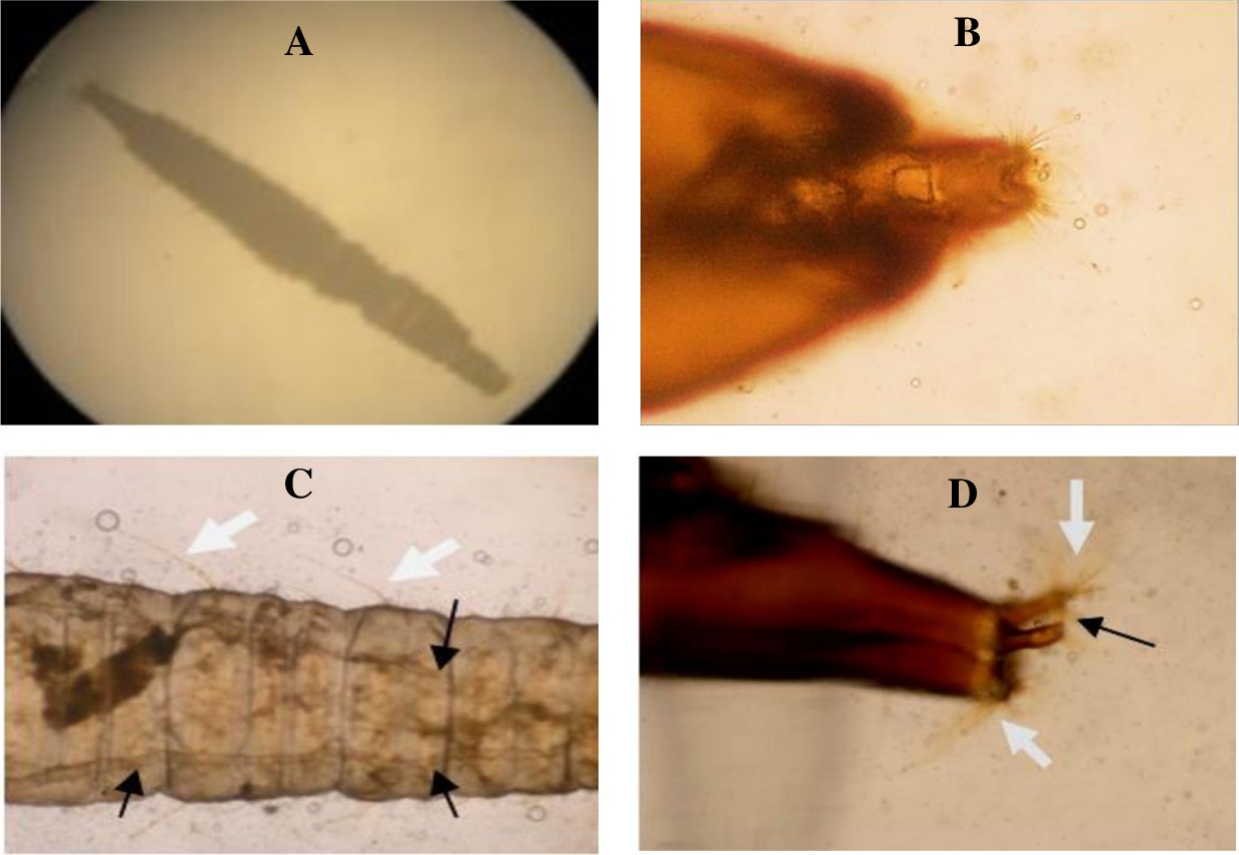
Light microscopic description of full-grown larvae of *Clogmia albipunctatus*. (A) The whole larva with an elongated, hairy, segmented body. (B) The triangular head, with 2-minute hairy antennae. (C) The abdominal segments, covered with long dark backwardly directed filiform setae (white arrows) and 2 breathing tubes extending along the length of the body (black arrows). (D) The last abdominal segment that tapers into two distinct respiratory tubules bearing the posterior spiracles apically (black arrow), which are guarded by dorsal and ventral brushes of hair tufts (white arrows).

The bodies of the larvae consist of a head and eleven segments (3 thoracic and 8 abdominal). They are cylindrical, hairy (Fig 1A) with distinct triangular head with 2-minute hairy antennae and lack prologs (Fig 1B). The mouthparts are of chewing type. They have feathered processes along their body segments. Two breathing tubes appeared extending along the length of the body (Fig 1C) starting with a pair of anterior spiracles at the prothorax and ended by a pair of posterior spiracles, at the tip of the terminal segment.

The abdomen is ended with a respiratory siphon guarded by dorsal and ventral brushes of hair tufts (Fig 1D). Caudally, the siphon is cone-shaped, showing a spinose anal papilla.

### Scanning Electron microscopy

**Fig 2:**
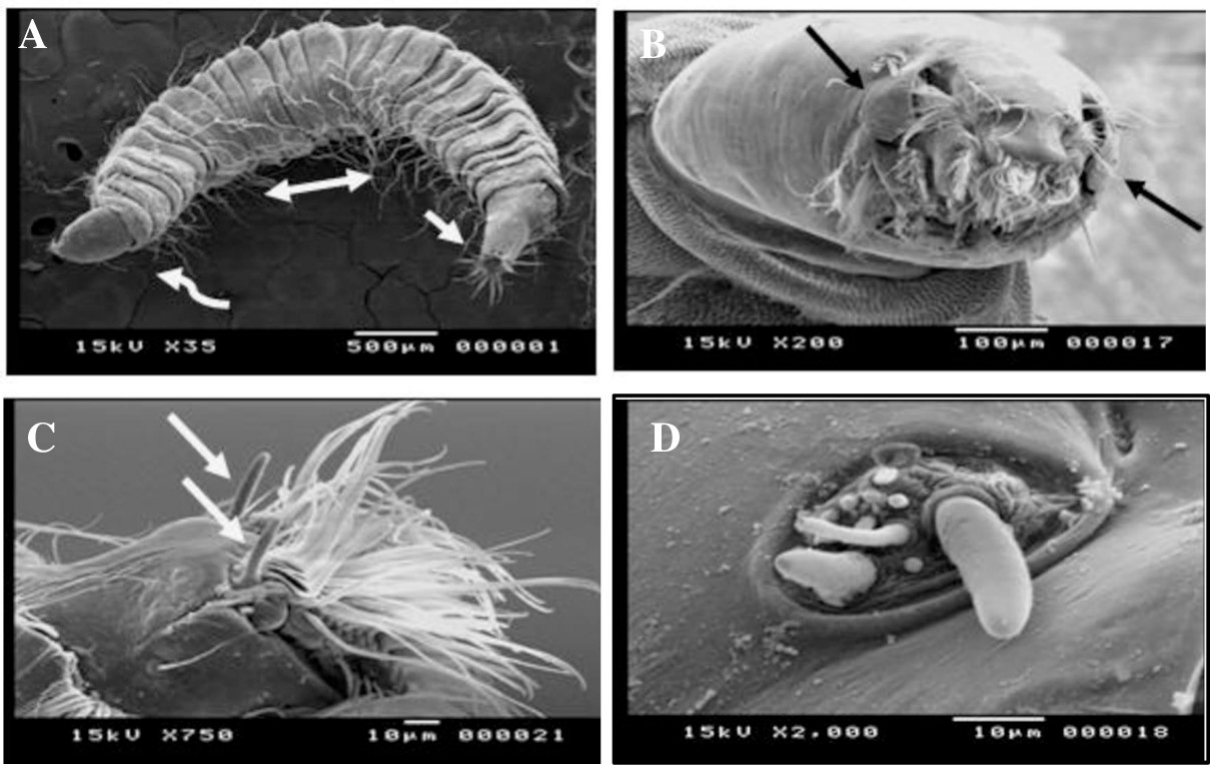
Scanning Electron microscopy of *Clogmia albipunctatus* whole body and the cephalic region. (A) The elongated, segmented, curved body with the anterior end (curved arrow), the posterior end (straight arrow) and numerous dorsal setae (double-headed arrow). (B) An anterior view of the cephalic region, showing the mouthparts with two lateral, small ear-like projections (black arrows). (C) The two-minute, unsegmented horny antennae (white arrows), each emerges from a basal socket. (D) The auricular opening with numerous simple papillae clustered together, in a well-developed basal ring with elevated edges.

The general body shape of the larvae is vermiform elongated and segmented. It has an annulated cuticle that tapers toward the end. (Fig 2A) The integument of the thoracic and abdominal segments is glabrous, while that of the head and the siphon is corrugated.

The dorsoventral view of the cephalic end clearly showed an ovate fully exposed non- retractable head capsule that is essentially smooth and bent ventrally. The most prominent features of anteroposterior view (frontal aspects) of the cephalic region (Fig 2B) are the labral brush with long setae and the two lateral small ears like structures lateral to both openings.

The antennae are minute, horny, unsegmented and borne on the anterior lateral borders of the head. Each antenna has a basal socket (Fig 2C).

The mandibles oppose each other in a horizontal plane. The maxillary palp complex is evident as five papillae more or less arranged in a row. The auricular opening presenting numerous simple papillae, with blunt tips, clustered together in a group that equipped with a well-developed basal ring with elevated edges (Fig 2D).

**Fig 3:**
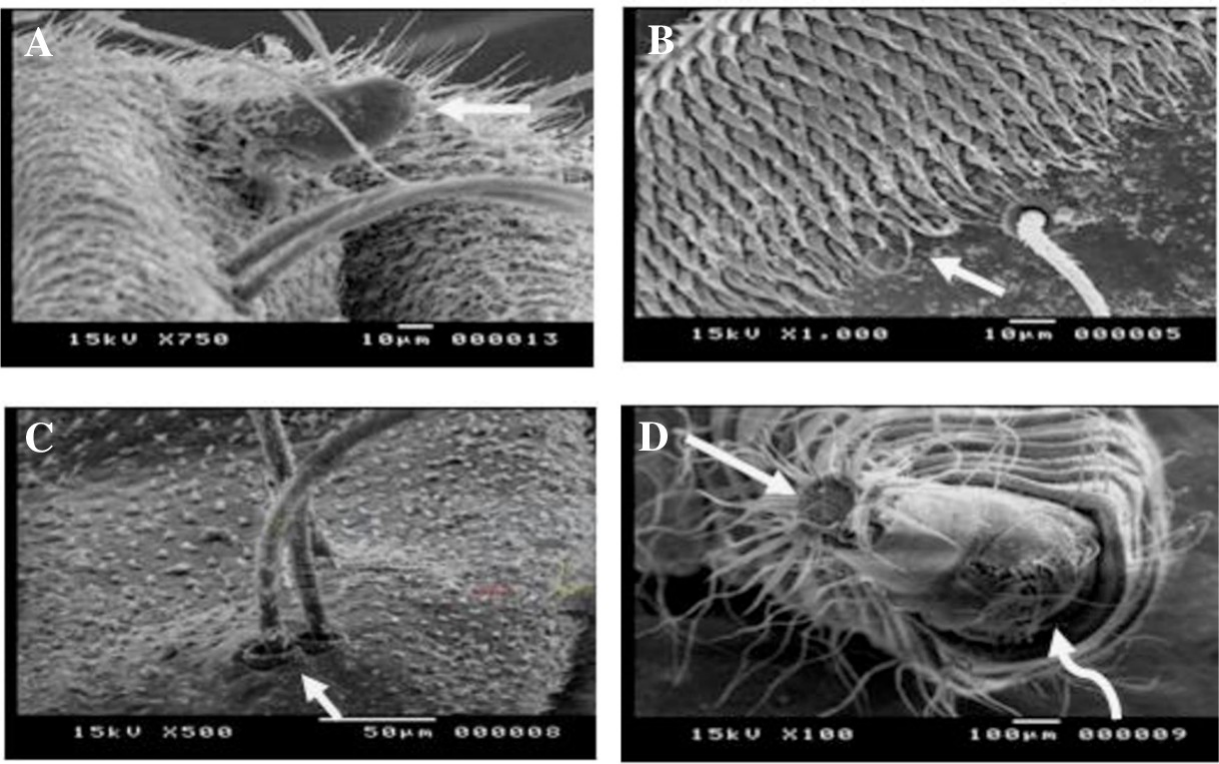
Scanning Electron microscopy of *Clogmia albipunctatus* body segments and the caudal end. (A) A nipple shaped anterior spiracle inserted laterally on the anterior annulus of the prothorax, with a solitary pore (white arrow). (B) A dorsal view of thoracic sensory spination with heavily packed tooth-like scales, arranged in several rows, each terminates sharply with a single bristle (white arrow). (C) All setae protrude individually from a hollow basal pocket (white arrow) with small barbs coming off the shaft. (D) The ventral surface of the siphon with the posterior spiracles opening at the apex of the respiratory tube (straight arrow), and a ventrally located anal papilla (curved arrow).

Anterior spiracles are nipple shape being inserted laterally on the anterior annulus of the prothorax. With higher magnification, it appeared situated at the top of a globular bulge with a solitary pore (Fig 3A).

While looking at the dorsal view of thorax, sensory spination appeared consisted of heavily packed scales arranged in several rows per segment. Closely viewed, they appeared tooth-like with sharp termination and each bearing a single bristle (Fig 3B). The filiform setae appeared very similar to crustacean serrate setae. Along their length was small barbs coming off the shaft and directed towards the pointed distal end. All setae protrude individually from a hollow basal pocket (Fig 3C).

The dorsal abdomen showed irregular rows of minute rose- thorn shaped spicules and numerous setae similar to those of the thorax.

Upon the dorsal and ventral surfaces of the caudal end of the maggot, the siphon is short and conical, longer than broader, with smooth cuticle and few scattered simple setae. The posterior spiracles sited on the apex of the respiratory tube on the siphon. It is surrounded by a radial sun ray pattern of hairs (Fig 3D).

On the terminal abdominal segment, there were the large bulging, paired anal papillae, with apical and lateral setae together with radiating scales.

### Molecular identification

**Fig 4:**
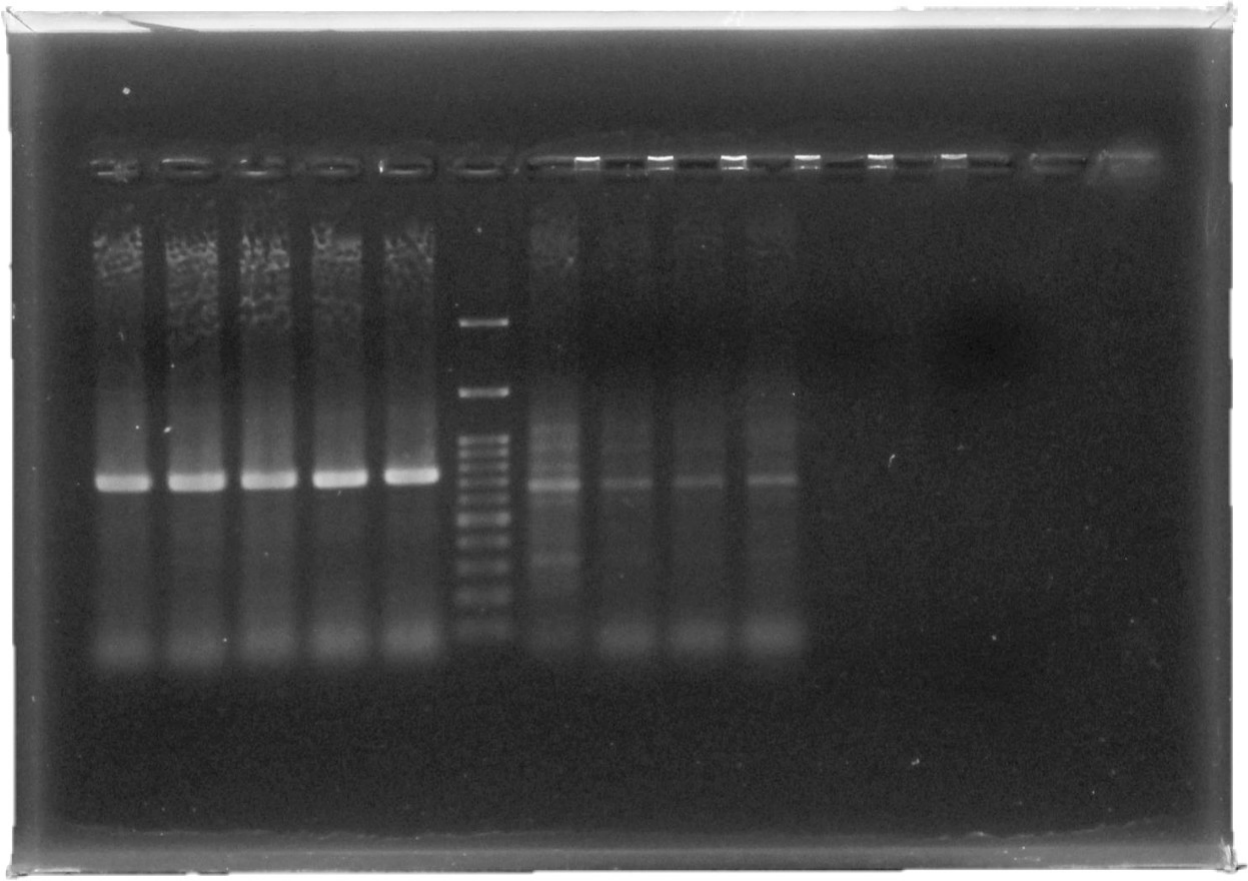
Molecular identification of DNA targeting two mitochondrial fragments of (cytB), NADH1 and 16S genes. With the agarose gel electrophoresis (1.5%), Lanes 1–5 are samples of tested isolates with an amplified product of 788 bp using primer pair CB-J-11338 and N1-N-12051, Lane 6: 100 bp DNA ladder. Lanes 7–10 are samples of tested isolates with an amplified product of 677using primer pair N1-J-12248 and LR-N-12866Lane 11: control negative (no template).

To confirm the diagnosis, molecular identification was done to clarify the larval species. All the procedures were conducted as previously described in the methodology sector. The DNA products were subsequently used for gel electrophoresis. Fig 4 shows the result of the PCR analysis (Fig 4).

As the sequences from our samples were all identical, resulting in the submission of only one sequence to GenBank.

Genetic sequencing was performed to prove the PCR result and to illuminate the larval species. After homology searching with the Nucleotide BLAST in the (NCBI) database (http://blast.ncbi.nlm.nih.gov/Blast.cgi). The sequence matched mainly *Clogmia albipunctatus* isolate 2 cytochrome b gene, under the accession number (JQ767023.1) with a query cover of 99%.The alignments displayed the following: score = 604 bits (327), expect 9e-169, identities = 329/331(99%), gaps = 0/331 (0%), and strand = Plus/Plus. The obtained sequence of Cytochrome B (cytB) gene was aligned with a reference sequence downloaded from Gen Bank (https://www.ncbi.nlm.nih.gov/nuccore/AB907184.1).

**Fig 5:**
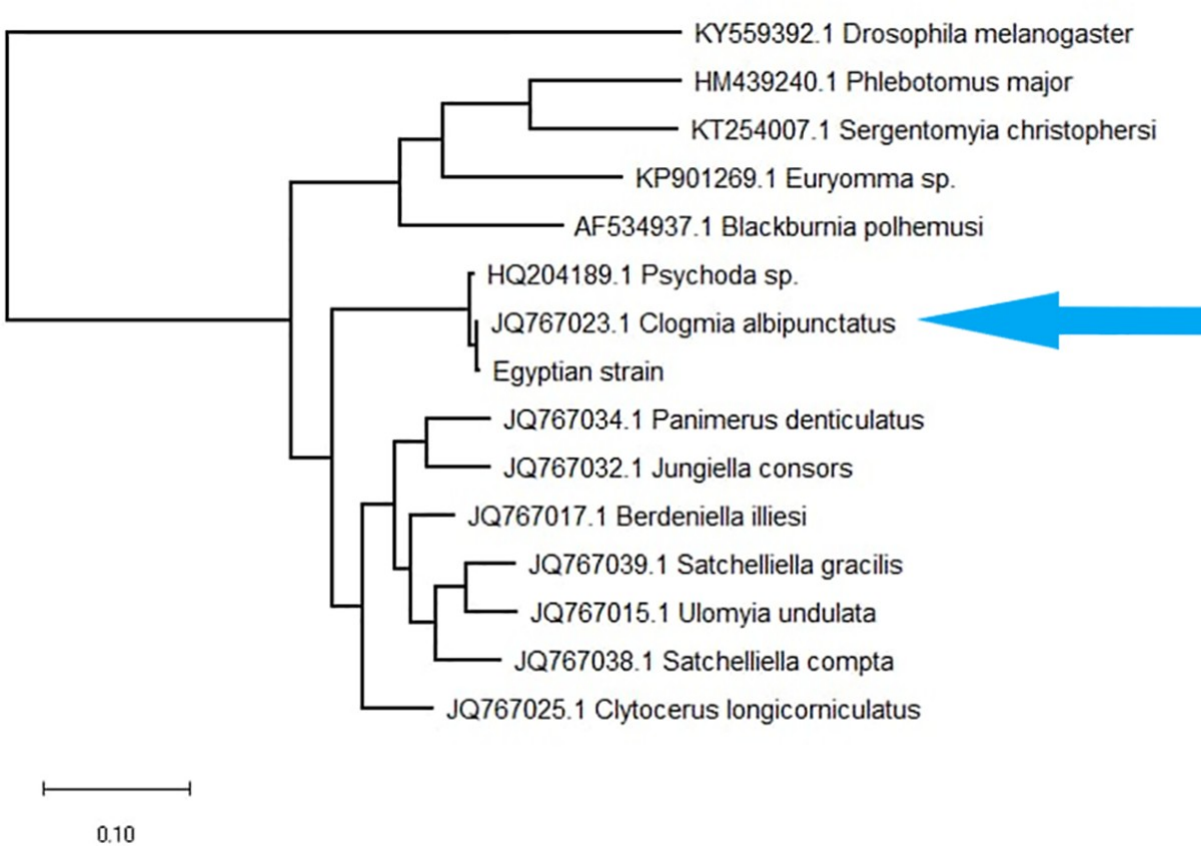
Evolutionary analysis by Maximum Likelihood method: The evolutionary history was inferred by using the Maximum Likelihood method. The tree with the highest log likelihood (-935.35) is shown. Initial tree(s) for the heuristic search were obtained automatically by applying Neighbor-Join and BioNJ algorithms to a matrix of pairwise distances estimated using the Maximum Composite Likelihood (MCL) approach and then selecting the topology with superior log likelihood value. The tree was drawn to scale, with branch lengths measured in the number of substitutions per site. This analysis involved 11 nucleotide sequences. There was a total of 420 positions in the final dataset. Evolutionary analyses were conducted in MEGA X.

Phylogenetic analysis was used by comparing of sequences that were downloaded from GenBank (KY559392.1, HM439240.1, KT254007.1, KP901269.1, F534937.1, HQ204189.1, JQ767023.1, JQ767034.1, JQ767032.1, Q767017.1, JQ767039.1, JQ767015.1, JQ767038.1, and JQ767025.1 (S1 Table). MUSCLE with default parameters was used for multiple sequence alignment. The phylogenetic tree was reconstructed using the maximum likelihood (ML) algorithm implemented in MEGA_X_10.0.4_win64 and edited with Figtree v1.4.2. The robustness of clusters was assessed by bootstrap values using 1,000 replicates. As the results show in Fig 5, our sequence was clustered with *C*. *albipunctatus* 'isolate reference sequence (Fig 5).

## Discussion

Myiasis is an infestation of human tissues and other vertebrates by eggs and larvae of flies of Diptera species. Urogenital myiasis develops when the fly larvae lay their eggs in the urogenital canal. A detailed history and appropriate laboratory tests should be performed, otherwise, this will be misdiagnosed as ureter stones and can lead to urinary complications [18].

Urinary myiasis usually occurs in low socioeconomic levels with overcrowding, inadequate ventilation, and poor sewage systems [19]. This was matched with our patients who were recorded to live in bad sanitation and poor hygienic measures. We speculated that patients of the current study were infected through urinating into unsanitary toilets or sleeping at night in warm weather without a covering.

Almost all of the reported cases of urogenital myiasis were caused by members Psychodidae family, which live mostly in the damp bathrooms [12]. Although human urinary myiasis is rare, many cases of myiasis with Psychoda spp. were reported all over the world in the last few years, at morphological and molecular bases [1] [12]. However, in Egypt, cases were reported only at a morphological level [2] [7] and this is the first report of urinary myiasis with *Clogmia albipunctatus*, in Egypt, at a molecular base.

In our study, with the identification of the larvae recovered from the urinary samples, they were found to be members of family Psychodidae. After comparing all their morphological characters to several literatures [19], they were identified as the fourth stage larva.

Proper identification of the species causing the myiasis is very important before treatment since not all types of myiasis are benign [20]. The present study used an ordinary microscopic identification of the larvae. However, SEM was carried out as a supportive method to illustrate some detailed features that may be more helpful in species identification. SEM has revealed the ultrastructure of different parts of the larvae such as the cephalic region, sensory structures, thoracic and abdominal spines, the respiratory spiracles and the pattern and distribution of sensory spination on the ventral and dorsal surfaces of the body segments and the distribution of the setae.

As reported from previous studies [21] [22], the larvae of the family Psychodidae are amphipneustic. They have a pair of thoracic and abdominal spiracles. The aquatic larvae of Psychodinae have the opening of the post abdominal spiracles at the apex of a respiratory siphon to adapt the aquatic mode of life, which is a good agreement with our findings. Moreover, in the current study, the anterior spiracles appeared situated at the top of a globular bulge with solitary pore. Such localization may favor the contact between the spiracles and the air as previously reported by Yones et al. [11].

Larvae of *C*. *albipunctata* could be differentiated from other members of genus Psychoda such *as P*. *latreille* and *P*. *albipennis* by having a darker body coloration and twenty-six dorsal plates while other members of genus Psychoda are much lighter in color and the number of dorsal plates is less than twenty-six. Moreover, the siphon of P. *albipennis* is more slender and 7 to 8 times as long as broad [2]. The spination of the larval segment should be taken into consideration as this can be helpful in differentiation [23].

Accurate species identification of the moth fly is markedly important for pest control measures. However, it is difficult to identify the larvae at a specific level, based only on SEM [9], they were reported in the current study as Psychoda sp. Further molecular characterization was needed to reach the species identification.

Psychodidae is one of the current worldwide families lacking specific phylogenetic studies. Recently, the popularization of molecular identification has triggered the reconstruction of phylogenetic status for this group [17]. With the approach of molecular techniques, the PCR assay, which targets the nuclear and mitochondrial genes, has been recently used for molecular identification and phylogenetic analysis of insect species. The cytochrome b (cytb), the mt-COI and mt-COII, the NADH dehydrogenase subunit 1, and 28S and 16S ribosomal RNA gene regions were all used to determine relationships within the subfamily Psychodinae worldwide [9]. In this study, the mitochondrial gene regions used were Cytochrome B (cytB), NADH1 and 16S. With sequencing and phylogenetic analysis, our sequence was clustered with *C*. *albipunctatus* 'isolate reference sequence. Similarly, a study was carried out by Mokhtar et al. [6] who diagnosed a case of intestinal myiasis in Malaysia by a stereomicroscope as a Psychodid larva. Then they used the COI gene region by DNA barcoding that suggested the larvae were C. *albipunctatus*.

A case of urinary myiasis was also reported in a female Chinese patient, both morphologically and molecularly based analysis using the cytochrome oxidase subunit 1 (COX1) gene that revealed a fourth instar larva of *Telmatoscopus albipunctatus* [12].

Another study was done in Turkey, by Onder et al. [9] identified the molecular characterization of *C*. *albipunctatus* by DNA barcoding technique, based on mt-COI. To the best of our knowledge, no molecular-based studies have been conducted on moth flies in Egypt, up to date.

Nevertheless, our study had a limitation; as the samples were collected from a single province in Egypt. Other future researches are required, for different regions in Egypt and for various regions in the world, to judge the extent of the problem and its prevalence. Such studies should increase their taxon sampling and sequence more markers at both mitochondrial and nuclear levels for a better understanding of the relationship within Psychodinae. Lastly, clinicians should pay attention to the infection of *C*. *albipunctatus*.

## Conclusion

Overall, through combined morphological and molecular approaches, our patients were accurately diagnosed with having *C*. *albipunctatus* infestation. Such results provided the first set of data on the molecular identification and phylogenetic analysis of moth flies based on DNA barcoding in Egypt. Thus, it presents more understanding of these species epidemiology, the vectorial potentiality, and proper pest management practices.

## Supporting information

S1 Table Accession numbers of genes. (DOCX)
