# Supplementary material for: Morphological and initial molecular characterization of Clogmia albipunctatus larvae (Diptera: Psychodidae) causing urinary myiasis in Egypt
Source: PLoS Negl Trop Dis. 2019 Dec 23;13(12):e0007887. doi: 10.1371/journal.pntd.0007887 (PMC6927617; doi:10.1371/journal.pntd.0007887)
Supplement: S1 Table — (DOCX) [file pntd.0007887.s001.docx]

**Supporting information**

**S1 Table. Accession numbers of genes.**

| **Name** | **Accession numbers** | **Database** |
| --- | --- | --- |
| *Drosophila melanogaster* | KY559392.1 | NCBI |
| *Phlebotomus major* | HM439240.1 | NCBI |
| *Sergentomyia christophersi* | KT254007.1 | NCBI |
| *Euryomma sp.* | KP901269.1 | NCBI |
| *Blackburnia polhemusi* | F534937.1 | NCBI |
| *Psychoda sp.* | HQ204189.1 | NCBI |
| *Clogmia albipunctatus* | JQ767023.1 | NCBI |
| *Panimerus denticulatus* | JQ767034.1 | NCBI |
| *Jungiella consors* | JQ767032.1 | NCBI |
| *Berdeniella illiesi* | Q767017.1 | NCBI |
| *Satchelliella gracilis* | JQ767039.1 | NCBI |
| *Ulomyia undulata* | JQ767015.1 | NCBI |
| *Satchelliella compta* | JQ767038.1 | NCBI |
| *Clytocerus longicorniculatus* | JQ767025.1 | NCBI |

**Abbreviation:** National Center for Biotechnology Information (NCBI)
